# Chloroplast characterization and phylogenetic relationship of *Cymbidium aloifolium* (Orchidaceae)

**DOI:** 10.1080/23802359.2019.1704656

**Published:** 2020-01-10

**Authors:** Juan Chen, Ming-Kun Chen, Qing-Dong Zheng, Shan-Hu Ma, Zhong-Jian Liu, Ye Ai

**Affiliations:** Key Laboratory of National Forestry and Grassland Administration for Orchid Conservation and Utilization at College of Landscape Architecture, Fujian Agriculture and Forestry University, Fuzhou, China

**Keywords:** *Cymbidium aloifolium*, chloroplast genome, Illumina sequencing, Orchid

## Abstract

*Cymbidium aloifolium* is an epiphytic orchid with high medicinal and ornamental value. In order to get a deeper understanding of *C. aloifolium*, we determined the complete chloroplast genome of *C. aloifolium* by Illumina sequencing data. The length of this genome is 157,328 bp, including a couple of inverted repeat (IR) regions of 26,829 bp, a large single-copy (LSC) region of 85,793 bp, and a small single-copy (SSC) region of 17,877 bp. The chloroplast genome comprised of 139 genes, including 78 protein-coding genes, 38 tRNA genes, and 8 rRNA genes. In addition, the phylogenetic analysis based on 17 chloroplast genomes of Orchidaceae indicated that *C. mannii* was closely related to *C. aloifolium*. This study will provide more valuable information for the classification and phylogenetic research of *Cymbidium* genus.

*Cymbidium aloifolium* is an epiphytic orchid widely distributed in tropical and subtropical regions. It often grows on the large branches or trunks in forests, and cliffs along streamsides and valleys (Liu et al. 2009). *C. aloifolium* has great horticultural value with leathery green leaves and long pendant inflorescences, and it is often used as a hybrid parent for the breeding of new varieties (Deb and Pongener 2011). Besides, *C. aloifolium* is used in traditional medicine with antinociceptive and anti-inflammatory activity to treat various human diseases (Howlader et al. 2011; Shah et al. 2019). Unfortunately, *C. aloifolium* has become an endangered species due to the indiscriminate collection and habitat loss (Pradhan et al. 2013). There were many studies on the tissue culture of *C. aloifolium* (Hossain et al. 2009), but little research has been done on genetic information. Therefore, we established the complete chloroplast genome sequence of *C. aloifolium*. Our work will not only promote further research and protection of *C. aloifolium*, but also provide more valuable information for species classification and phylogenetic relationship in *Cymbidium* genus.

In this study, fresh leaves of *C. aloifolium* were sampled from Caoguoshan Mountain, Malipo Country, Wenshan Prefecture, Yunnan Province, China (23°5′26.61″N, 104°42′3.15″E). The voucher specimen was kept at the Herbarium of Fujian Agriculture and Forestry University (specimen code FAFU01837).

The total genomic DNA was extracted from fresh leaves by using TIANGEN DNA Extraction Kit (TIANamp Genomic DNA Kit, Beijing, China), and used to construct a library for sequencing with Illumina Hiseq 2500 platform and approximately 5.0 Gb of sequence data were generated. After removing adapters and low-quality reads by fastp software (Chen et al. 2018), the chloroplast genome was assembled using GetOrganelle (Ankenbrand et al. 2018) based on obtained sequence data. The genome was annotated using the software CpGAVAS (Liu et al. 2012), then adjusted by Geneious 8.0.4 (Kearse et al. 2012). Finally, the complete chloroplast genome sequence of *C. aloifolium* with gene annotated was submitted to GenBank with the accession number MN641752.

The chloroplast genome sequence of *C. aloifolium* is 157,328 bp in length. It contains a couple of inverted repeat (IR) regions of 26,829 bp, a large single-copy (LSC) region of 85,793 bp, and a small single-copy (SSC) region of 17,877 bp. Besides, the chloroplast genome contains 139 genes, including 78 protein-coding genes, 38 tRNA genes, and 8 rRNA genes. The overall GC content of the whole genome is 36.8% (LSC, 34.3%; SSC, 29.5%; IR, 43.3%).

To investigate the phylogenetic location of *C. aloifolium*, 10 complete chloroplast genomes of Cyrtopodiinae were used to construct a phylogenetic tree, and 6 complete chloroplast genomes from other genus in Orchidaceae were used as outgroup. All the sequences were downloaded from GenBank, and aligned using MAFFT v.7 (Katoh and Standley 2013). Then, a maximum likelihood tree was constructed by using RAxML (Stamatakis 2014) with 1000 bootstrap replicates. The ML tree analysis indicated that *C. aloifolium* was most closely related to *C. mannii* with 100% bootstrap support (Figure 1).

**Figure 1.**
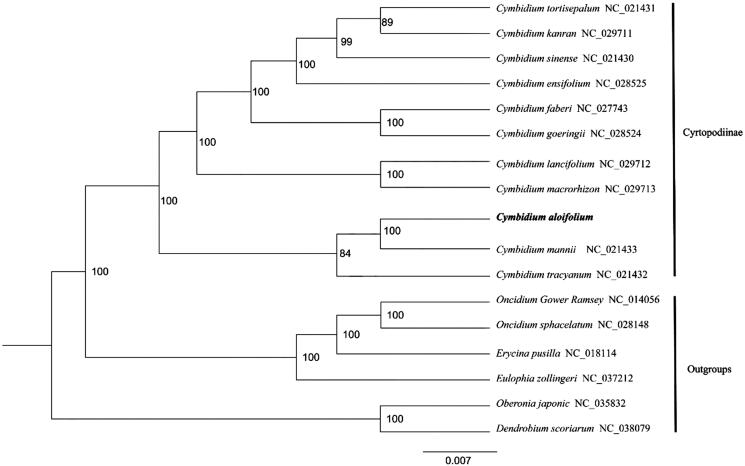
The maximum likelihood tree based on 17 complete chloroplast genome sequences of Orchidaceae, and the position of *C. aloifolium* is shown in bold.
